# Co-occurrence of Peutz-Jeghers syndrome and unilateral multicystic dysplastic kidney: a case report

**DOI:** 10.1186/s12882-025-04340-8

**Published:** 2025-07-17

**Authors:** Yaqing Liu, Sunhe Hu, Yihan Gan, Yulan Fang

**Affiliations:** 1 Department of Pediatrics, First Affiliated Hospital, Gannan Medical University, No. 128, Jinling West Road, Zhanggong District, Ganzhou, 341000 JiangXi China; 2 Gannan Medical University, No. 1, Medical College Road, Ganzhou, 341000 China

**Keywords:** Peutz-Jeghers syndrome, Multicystic dysplastic kidney, *STK11*, *LKB1*, Case report

## Abstract

**Background:**

This case study aimed to evaluate the co-occurrence of Peutz-Jeghers syndrome (PJS) and unilateral multicystic dysplastic kidney (MCDK) disease through clinical and genetic analysis of a patient with both conditions.

**Case presentation:**

A 13-year-and-7-month-old female patient presented with typical features of PJS, including pigmented polyposis of the gastrointestinal tract, dark pigmented spots on the skin, and a history of intestinal intussusception. Genetic testing revealed a pathogenic mutation in the serine/threonine protein kinase 11 (*STK11)* gene c.843del frameshift variant (p.L282Sfs*5), characterized by guanine deletion at position 843 resulting in leucine-to-serine substitution at residue 282, complete alteration of downstream amino acid sequence, and premature translation termination. In addition, she also presented with MCDK.

**Conclusion:**

This case reveals a potential novel role of STK11 in renal embryogenesis, expanding its phenotypic spectrum and challenging the conventional paradigm that STK11 mutations solely confer tumor predisposition. Through comprehensive literature review, we propose three mechanistic hypotheses - “metabolo-developmental coupling disruption”, “ciliopathy-like pathogenesis”, and “epigenetic modulation of developmental plasticity” - providing new molecular insights into congenital renal anomalies. These findings warrant systematic renal evaluation in PJS patients and exploration of metabolism-targeted therapeutic strategies.

**Clinical trial number:**

Not applicable.

## Background

Peutz-Jeghers syndrome (PJS), also referred to as pigmented polyposis of the gastrointestinal tract or black spot polyposis, is an autosomal dominant hereditary disorder (OMIM 175200) characterized by the presence of multiple benign hamartomatous polyps within the gastrointestinal tract, as well as distinctive pigmentation of the skin and mucous membranes. Furthermore, individuals with PJS exhibit an elevated risk for both gastrointestinal and non-gastrointestinal malignancies [1]. PJS is considered a rare condition, with an estimated population incidence ranging from 1:200000 to 1:8000 [2], and it does not demonstrate a significant gender predilection. Approximately half of the PJS cases have a familial history. However, sporadic cases and skip-generation inheritance were also observed. Multicystic dysplastic kidney (MCDK) is a common renal cystic disorder in children, characterized by non-communicating cysts of varying sizes within undifferentiated and immature tissues. The incidence of unilateral MCDK is approximately 1 in 4,300 live births and is more common in males [3]. MCDK may present as an isolated defect or as a part of hereditary multi-organ syndromes [4], including Zellweger syndrome (Online Mendelian Inheritance in Man (OMIM) 214100), branchio-oto-renal syndrome (OMIM 113650), Bardet-Biedl syndrome (OMIM 209900), and Eagle-Barrett syndrome (OMIM 118494). This case study aimed to retrospectively analyze the clinical and genetic findings of a female patient diagnosed with both PJS and MCDK to explore the potential genetic links between the two conditions and to better understand this condition.

## Case description

### Case presentation

The proband, a female, aged 13years and 7 months, complained of abdominal pain for 2 h and was admitted to the hospital on July 24, 2024. During admission, the patient experienced vomiting but did not exhibit diarrhea, bloody stool, or fever. Regarding previous medical history, at six and a half years old, the patient underwent surgical intervention for secondary intussusception caused by intestinal polyps. The patient had a history of several urinary tract infections. At the age of 3, during hospitalization for a common cold, ultrasound examination failed to detect the right kidney, and it was consistently diagnosed as “right renal agenesis”. The patient’s parents are not consanguineously related, and there is no reported family history of similar conditions.

Following admission to the hospital, the patient underwent several investigations, including a physical examination, laboratory examinations, endoscopy, radiological examinations, histopathological examinations and gene testing. The results were as follows.

### Physical examination

**Fig. 1:**
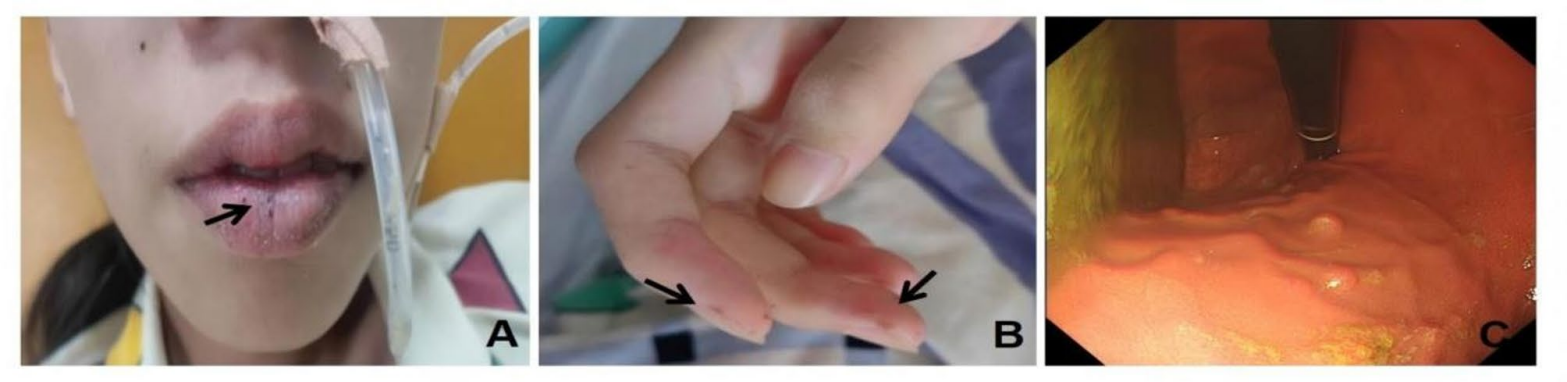
Dark pigmented spots on the skin at the peripheries of the lips (**A**) and fingers (**B**); Gastroscopy showed multiple gastric polyps (**C**)

The physical examination revealed a body temperature of 36.1 °C, a pulse rate of 90 beats per minute, a respiration rate of 21 breaths per minute, and a blood pressure of 98/56 mmHg (1 mmHg = 0.133 kPa). The patient had a height of 157 cm and a weight of 33 kg, indicating a slightly underweight body habitus. Examination of the mucous membranes and skin revealed no jaundice, petechiae, or swelling of superficial lymph nodes. However, dark pigmented spots, ranging from 1 mm to 3 mm in diameter, were observed on the upper and lower lips and the distal phalanges of both hands (Fig. 1). A cardiopulmonary examination revealed no abnormalities. The abdomen was flat and soft, with a 4 cm longitudinal surgical scar on the right abdominal wall. There was no evidence of gastrointestinal distention or peristaltic waves, and no masses were palpable. Mild tenderness was noted around the umbilical region, with no rebound tenderness or tenderness at McBurney’s point. The Murphy’s sign was negative, and the bowel sounds were normal. There was no edema in the lower extremities, and the neurological examination was unremarkable.

### Laboratory examinations

The complete blood count results showed a white blood cell count of 8.83 × 10^9/L, hemoglobin level of 110 g/L, and platelet count of 525 × 10^9/L. Blood biochemistry analysis revealed an alanine transaminase (ALT) of 9 U/L, aspartate transaminase (AST) of 15 U/L, albumin level of 32 g/L, urea nitrogen of 3.8 mmol/L, and creatinine level of 40 µmol/L. The uric acid and electrolyte levels were within normal ranges.

### Endoscopy and other radiological findings

**Fig. 2:**
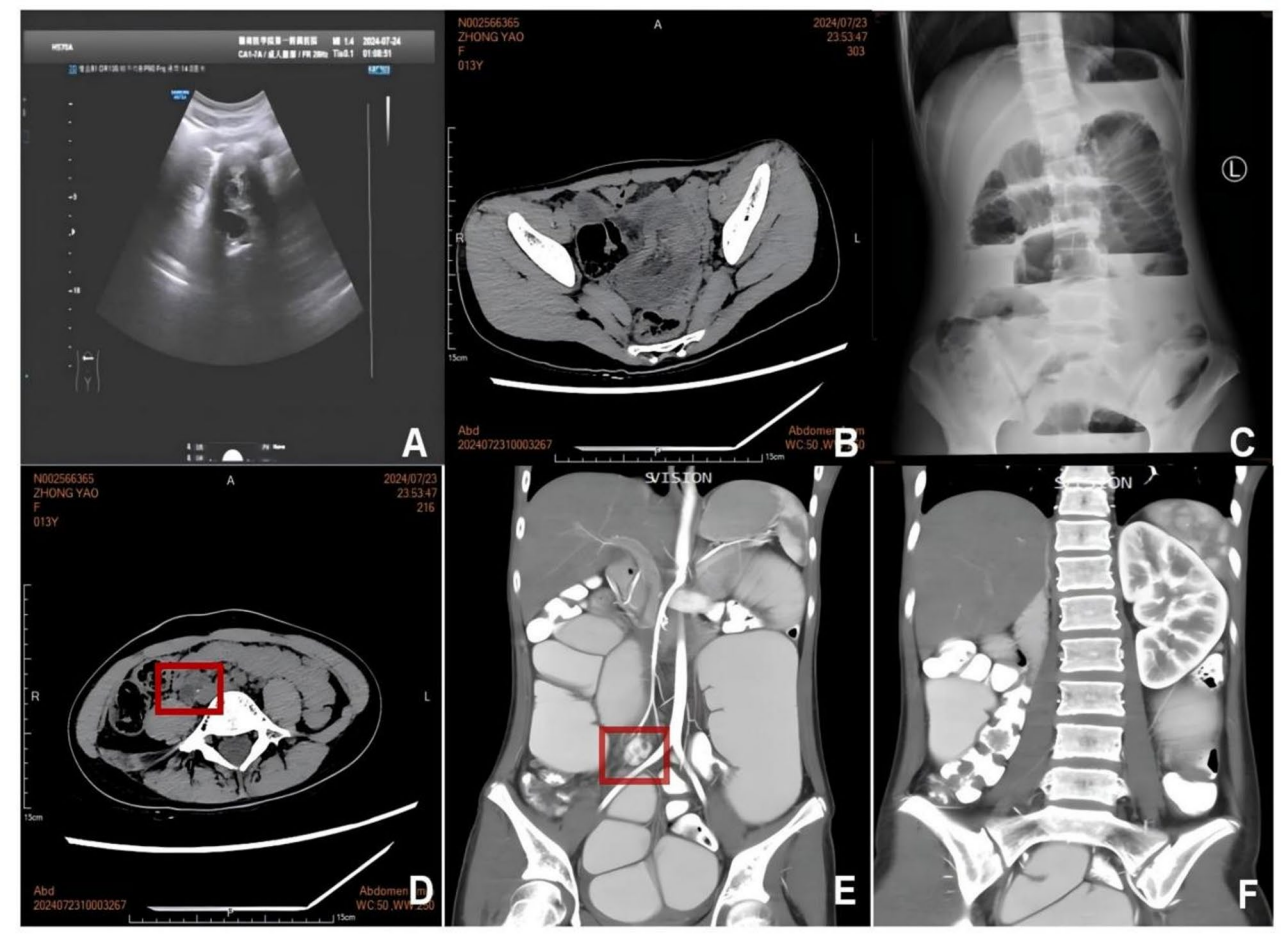
Imaging findings. Ultrasound (**A**) and Abdominal computed tomography (**B**) showed intussusception; Plain abdominal X-ray showed intestinal obstruction (**C**); Abdominal CT showed a complex cystic-solid mass adjacent to the right psoas muscle (marked by red box) (**D**); Contrast-enhanced abdominal computed tomography angiography demonstrated an atrophic right kidney with multiple cysts (marked by red box) (**E**) and compensatory enlargement of the left kidney (**F**)

Electronic gastroscopy identified a gastric polyp and atrophic gastritis with erosion, primarily in the antrum (Fig. 1). Abdominal ultrasound and abdominal computed tomography scan showed intussusception with effusion. Gynecological ultrasound revealed no significant abnormalities in the uterus and adnexa. Abdominal X-ray demonstrated scattered gas shadows within the intestines, localized intestinal gas dilatation, and multiple gas-liquid levels in the middle and lower abdomen, indicating incomplete intestinal obstruction. An abdominal computed tomography scan revealed small intestinal obstruction, abdominal and pelvic effusion, and absence of the right kidney, slight enlargement of the left kidney, and a cystic-solid lesion in the right psoas paraspinal region. The contrast-enhanced abdominal computed tomography angiography with 3D reconstruction revealed a slender right renal artery, significant volume reduction of the right kidney at the L5 vertebral level with multiple non-enhancing hypodense lesions, and compensatory left renal hypertrophy (Fig. 2).

### Histopathology

**Fig. 3:**
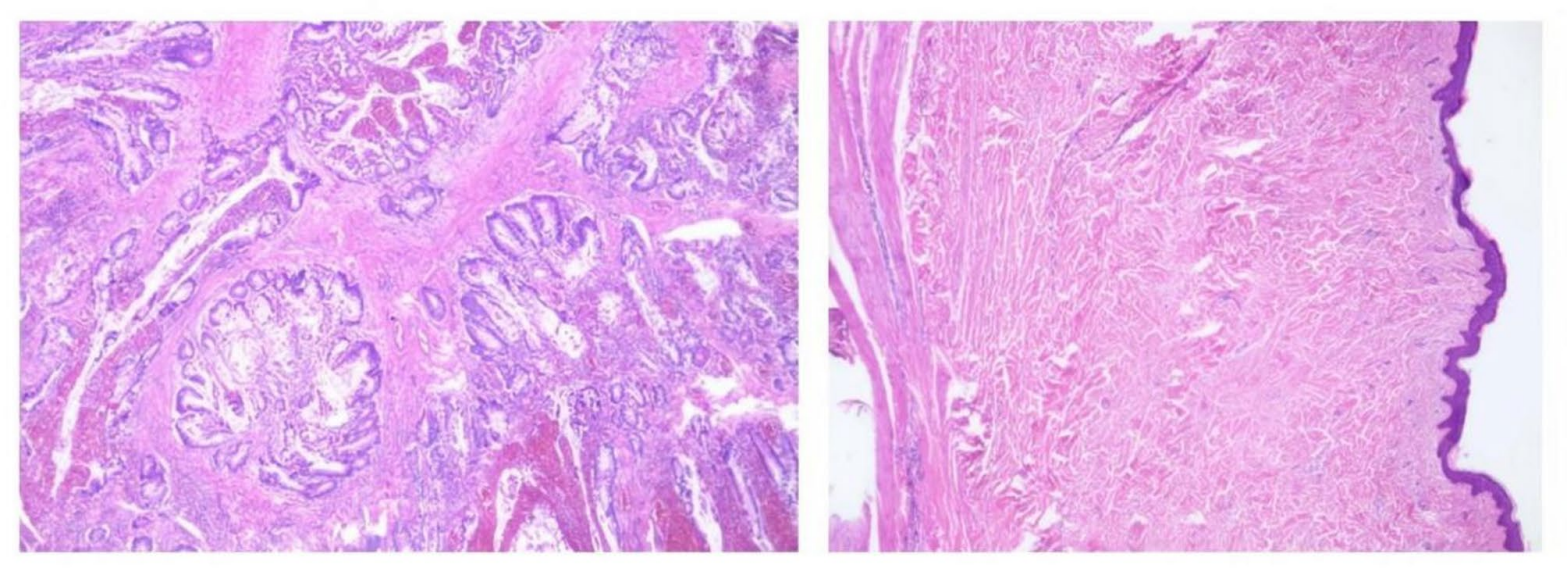
Histological image of a small intestinal hamartomatous polyp (HE×100)

An intestinal tissue biopsy was performed. The histopathological analysis of the intestinal wall (Fig. 3) showed hemorrhagic and necrotic changes in the mucosal layer, along with a loose and edematous submucosal layer. The blood vessels throughout the intestinal wall were dilated and congested. Histopathological analysis of the intestinal polyp showed a benign hamartomatous polyp, associated with hemorrhage and necrosis.

### Gene testing

**Fig. 4:**
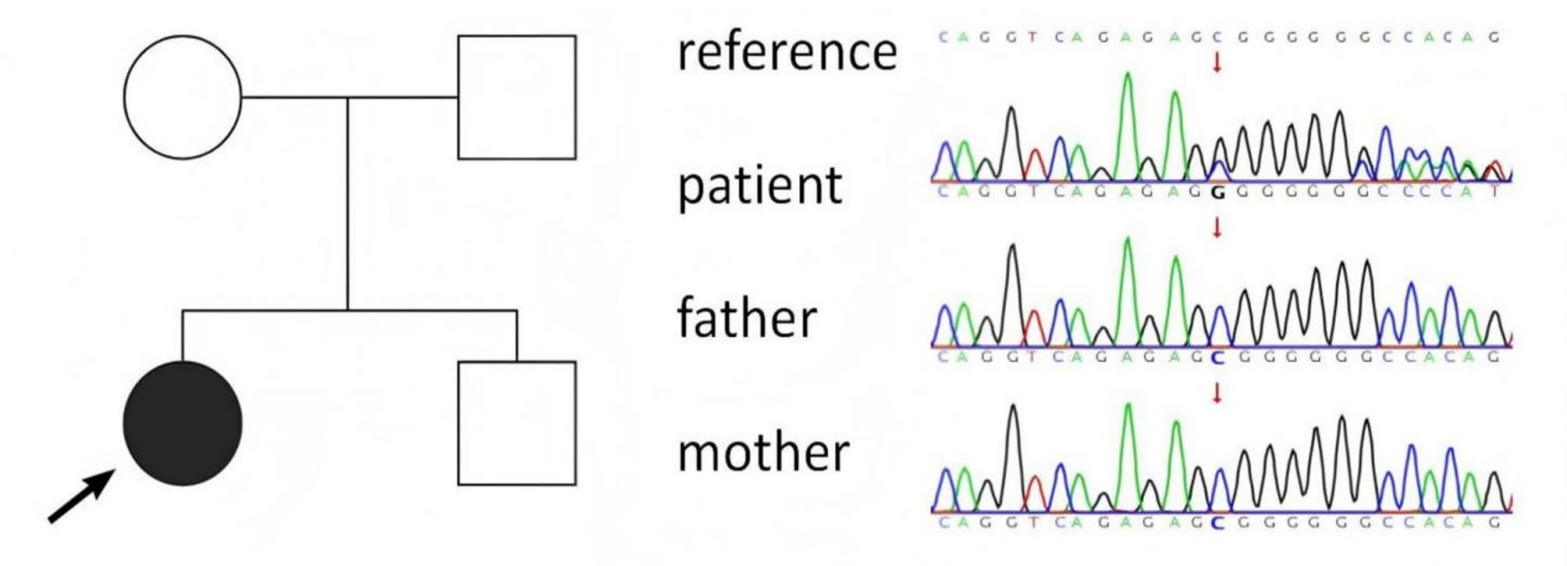
Pedigree chart and sanger sequencing verification

Whole-exome sequencing revealed a deletion of nucleotide 843G in exon 6 of the serine/threonine protein kinase 11 (*STK11)* gene, leading to a frameshift mutation, a premature stop codon, and the production of truncated proteins, thereby causing functional alterations in the protein. Sanger sequencing confirmed the presence of a heterozygous variant, c.843del (p.L282Sfs*5), in the patient’s *STK11* gene (Fig. 4). The parental locus was identified as wild type, indicating a de novo mutation consistent with an autosomal dominant inheritance pattern. Based on the American College of Medical Genetics and Genomics (ACMG) guidelines, the L282Sfs*5 mutation in the *STK11* gene was identified as pathogenic. The patient’s immediate family, including the parents, younger brother, and other close relatives, exhibited no similar conditions, genetic disorders, or pertinent symptoms. Consequently, due to the identification of a spontaneous mutation in the patient, genetic testing was not extended to family members beyond the parents.

### Treatment and follow-up

On July 24, 2024, the patient underwent laparoscopic exploration, reduction of intussusception, lysis of intestinal adhesions, and partial resection of the small intestine. Following the procedure, the patient had a right abdominal debridement and skin flap plasty to ensure proper healing and prevent infections within the surgical wound. Postoperatively, the patient experienced recurrent upper abdominal pain, and diagnostic imaging indicated intestinal obstruction. The patient was subsequently transferred to a higher-level pediatric surgery department, where a diagnosis of low-grade partial intestinal obstruction was made, prompting a partial small bowel resection on September 3, 2024. Due to poor abdominal wound healing, the patient required one month of hospitalization before gradual recovery and eventual discharge. The patient received medical nutrition therapy (MNT) following discharge. At the 6-month follow-up evaluation, a 10 kg weight gain was observed. Her menarche occurred at age 14, and since that time she has maintained regular menstrual cycles.

## Discussion

PJS is a rare autosomal dominant disorder characterized by the presence of hamartomatous polyps in the gastrointestinal tract, alongside distinctive pigmentation of the skin and mucosa. In some instances, patients with PJS exhibit renal anomalies, such as renal dysplasia, renal cysts, and renal cell carcinoma. However, to our knowledge, no cases of MCDK have been reported in patients diagnosed with PJS with *STK11* mutations [5, 6]. This report introduces a novel phenotype that may offer insights into the genetic correlation between PJS and MCDK.

MCDK is a relatively prevalent congenital anomaly of the urinary system, typically identified during fetal ultrasound screenings and occasionally detected incidentally during postnatal medical examinations. The precise etiology of MCDK remains incompletely understood. Current hypotheses suggest it may result from either abnormal inductive interactions between the ureteric bud and metanephric mesenchyme during renal embryogenesis or from a fetal urinary tract obstruction occurring in utero [7, 8]. However, other genetic factors may also play a role in the development of renal abnormalities during kidney morphogenesis. One study analyzing coding exons of congenital anomalies of the kidney and urinary tract (CAKUT)-associated genes in a CAKUT cohort identified correlations between MCDK and mutations in *CHD1L*,* ROBO2*,* HNF1B*,* and SALL1* [9]. In a separate investigation utilizing array comparative genomic hybridization (aCGH), three distinct pathogenic genetic variants conforming to inheritance patterns were identified in a cohort of 10 unrelated pediatric MCDK cases: a deletion at 7p14.3; a duplication at 16p13.11p12.3, and monosomy X for a female patient [10]. The presence of these mutations supports the role of genetic factors in the development of MCDK.

**Fig. 5:**
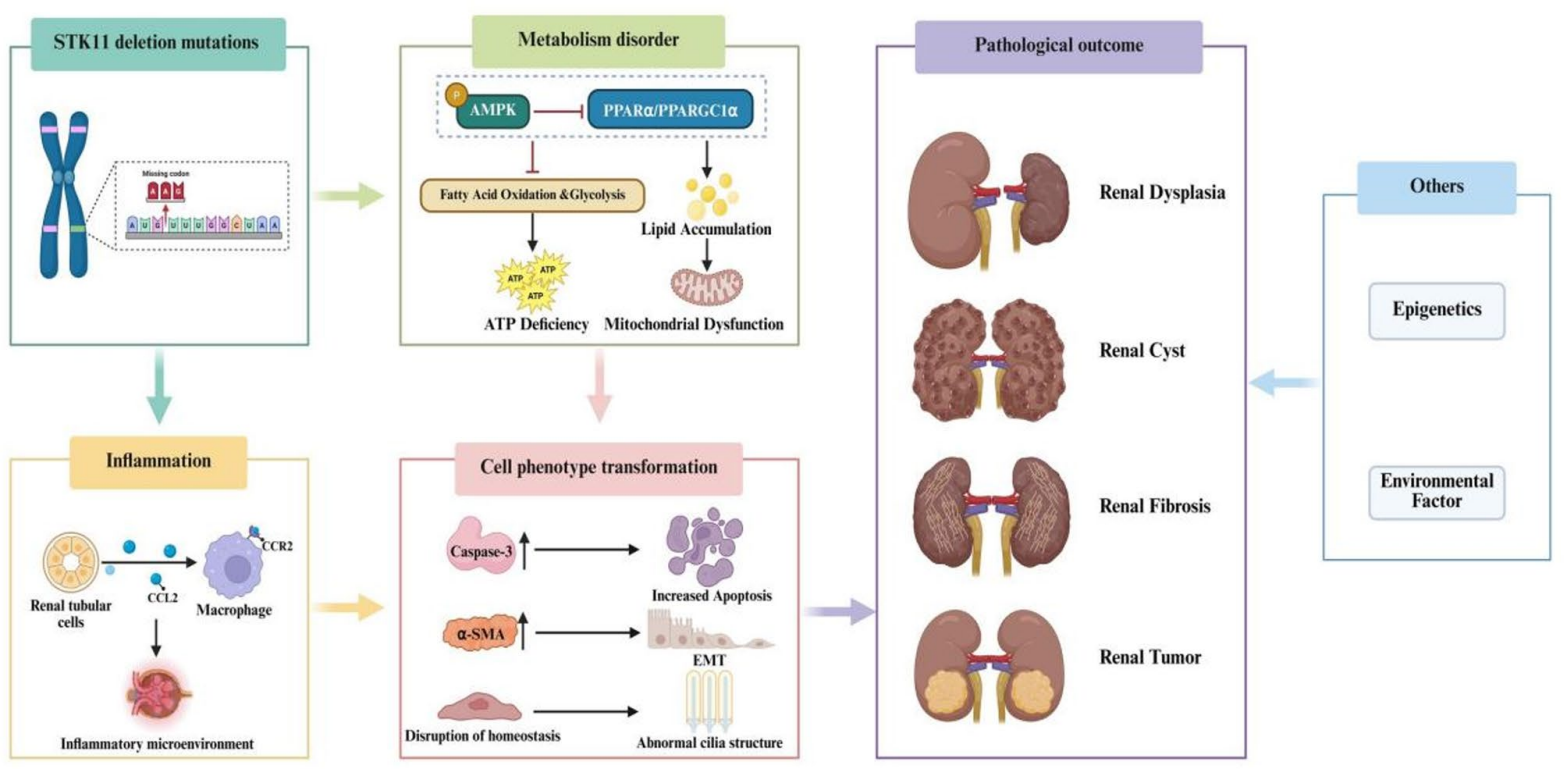
Pathophysiological changes induced by *STK11* deletion mutation

The most prevalent pathogenic gene linked to PJS is the *STK11* gene, also referred to as liver kinase B1 (*LKB1*), which is located on chromosome 19p13.3. This gene encodes a serine/threonine kinase [11, 12] that regulates the activity of members of the AMP-activated protein kinase (AMPK) family, thereby influencing critical cellular processes such as cell polarity, metabolism, and apoptosis, which can ultimately lead to abnormalities in cell growth and differentiation [13]. Prior research has demonstrated that kidney-specific *STK11* deletion mutations can exacerbate renal fibrosis by disrupting cellular metabolic pathways [14, 15]. Furthermore, Han et al. [15] found that *STK11* expression is significantly reduced in individuals with chronic kidney disease compared to healthy controls. Their findings also revealed that specific deletion (*Ksp-Cre/Lkb1*^flox/flox^) of the *STK11* gene in cultured renal tubular epithelial cells led to reduced expression levels of key metabolic process regulators, including AMPK, peroxisome proliferator-activated receptor gamma coactivator 1 alpha (PPARGC1α), and peroxisome proliferator-activated receptor alpha (PPARα). The alteration of intracellular metabolism can lead to increased energy consumption in cells, decreased fatty acid oxidation and glycolysis, and the promotion of apoptosis and renal fibrosis. Treatment of *STK11*-deficient cells with AMPK or PPARα agonists has been shown to restore lipid oxidation defects and reduce apoptosis. These findings indicate that *STK11* gene mutations may play a significant role in the pathogenesis of kidney disease through their influence on metabolic processes. Furthermore, studies using mouse models have shown that deletion mutations in the *STK11* gene in renal tubules result in increased expression of the chemokine C-C motif ligand 2 (CCL2) in a cell-autonomous manner. This leads to the accumulation of C-C chemokine receptor type 2 (CCR2)-positive mononuclear phagocytes around the renal tubules. Such dysregulation of cellular homeostasis may contribute to the development of a ciliopathy phenotype [16]. These findings suggest that *STK11* mutations could adversely affect renal development during embryogenesis, potentially resulting in renal dysplasia, renal cysts, and renal tumors. Although there is currently no definitive evidence establishing a direct genetic link between PJS and unilateral multicystic dysplastic kidney, it is plausible that these two conditions may share certain genetic predispositions. Additionally, environmental factors and other genetic factors may also contribute to the common pathogenesis of these diseases (Fig. 5).

In the context of clinical management, during childhood and adolescence, complications associated with PJS include polyps, intussusception, intestinal obstruction, gastrointestinal bleeding, malnutrition, and developmental delay. Most of these patients require surgical intervention to address complications such as intussusception and intestinal obstruction. In adulthood, PJS is primarily characterized by an increased susceptibility to tumor development. Unilateral MCDK is generally considered a benign condition, with the affected kidney typically undergoing progressive involution over time [17]. The most rapid atrophy typically occurs within the first 2 to 3 years of life, and the involution generally ceases at a median age of 5.5 years [18]. The majority of MCDK cases demonstrate spontaneous regression without long-term complications. Therefore, conservative management remains the standard approach for most patients. While unilateral MCDK is generally considered to have low risks of hypertension and malignant transformation, a study revealed that 41% of patients still exhibit renal remnants with impaired glomerular filtration rate (GFR) after 10 years [19]. Therefore, conservative management of unilateral MCDK should be based on long-term follow-up surveillance. Furthermore, the potential interactions between coexisting PJS and MCDK and their collective impact on disease progression and clinical outcomes with age remain to be elucidated.

This case study has several limitations that have to be acknowledged. Since the patient did not undergo surgical resection of the dysplastic kidney, we were unable to obtain pathological results, and the brief follow-up period may impede a comprehensive understanding of the condition’s progression, the efficacy of treatments, and the potential development of complications. Additionally, the potential association between PJS and MCDK remains unclear, and their coexistence may be coincidental. Further cases are necessary to substantiate any potential link between PJS and MCDK and hence confirm that their coexistence is not coincidental.

## Conclusion

This case report presented a clinical and genetic analysis of a female patient diagnosed with PJS accompanied by MCDK. This case suggests the necessity for a renal evaluation in patients diagnosed with PJS. Future research is required to investigate the genetic linkages between PJS and other congenital anomalies to enhance the diagnostic and management strategies for these conditions and their associated complications.

## Data Availability

The datasets analysed during the current study are available in the Genome Variation Map repository, [Accession Number: GVM000956].

## Abbreviations

PJS: Peutz-Jeghers syndrome
MCDK: Multicystic dysplastic kidney
STK11: Serine/threonine protein kinase 11
CAKUT: Congenital abnormalities of the kidney and urinary tract
ECG: Electrocardiogram
ALT: Alanine transaminase
AST: Aspartate transaminase
CT: Computed tomography
ACMG: American College of Medical Genetics and Genomics
LKB1: Liver kinase B1
AMPK: AMP-activated protein kinase
PPARGC1α: Peroxisome proliferator-activated receptor gamma coactivator 1 alpha
PPARα: Peroxisome proliferator-activated receptor alpha
CCL2: Chemokine C-C motif ligand 2
CCR2: C-C chemokine receptor type 2
